# Investigation of Potential Gut Health Biomarkers in Broiler Chicks Challenged by *Campylobacter jejuni* and Submitted to a Continuous Water Disinfection Program

**DOI:** 10.3390/pathogens13050356

**Published:** 2024-04-26

**Authors:** Tilemachos Mantzios, Despoina E. Kiousi, Georgia D. Brellou, Georgios A. Papadopoulos, Vangelis Economou, Marili Vasilogianni, Elisavet Kanari, Evanthia Petridou, Ilias Giannenas, Guillermo Tellez-Isaias, Aglaia Pappa, Alex Galanis, Vasilios Tsiouris

**Affiliations:** 1 Unit of Avian Medicine, Clinic of Farm Animals, School of Veterinary Medicine, Aristotle University of Thessaloniki, 546 27 Thessaloniki, Greece; biltsiou@vet.auth.gr; 2 Department of Molecular Biology and Genetics, Faculty of Health Sciences, Democritus University of Thrace, 68 100 Alexandroupolis, Greece; dkiousi@mbg.duth.gr (D.E.K.); eliskanari@gmail.com (E.K.); apappa@mbg.duth.gr (A.P.); agalanis@mbg.duth.gr (A.G.); 3 Laboratory of Pathology, School of Veterinary Medicine, Aristotle University of Thessaloniki, 546 27 Thessaloniki, Greece; 4 Laboratory of Animal Husbandry, School of Veterinary Medicine, Aristotle University of Thessaloniki, 541 24 Thessaloniki, Greece; geopaps@vet.auth.gr; 5 Laboratory of Hygiene of Animal Food Products—Veterinary Public Health, School of Veterinary Medicine, Aristotle University of Thessaloniki, 541 24 Thessaloniki, Greece; boikonom@vet.auth.gr; 6 Pathobiology and Population Sciences, Royal Veterinary College, London NW1 0TU, UK; mvasilogianni21@rvc.ac.uk; 7 Laboratory of Microbiology and Infectious Diseases, School of Veterinary Medicine, Aristotle University of Thessaloniki, 541 24 Thessaloniki, Greece; epetri@vet.auth.gr; 8 Laboratory of Nutrition, School of Veterinary Medicine, Aristotle University of Thessaloniki, 541 24 Thessaloniki, Greece; igiannenas@vet.auth.gr; 9 Department of Poultry Science, University of Arkansas, Fayetteville, AR 72701, USA; gtellez@uark.edu

**Keywords:** biomarkers, gut health, *Campylobacter jejuni*, broilers, water disinfection, gut microbiota, gut histomorphology

## Abstract

The exploration of novel biomarkers to assess poultry health is of paramount importance, not only to enhance our understanding of the pathogenicity of zoonotic agents but also to evaluate the efficacy of novel treatments as alternatives to antibiotics. The present study aimed to investigate potential gut health biomarkers in broiler chicks challenged by *Campylobacter jejuni* and subjected to a continuous water disinfection program. A total of 144 one-day-old hatched broiler chicks were randomly allocated to four treatment groups with four replicates each, according to the following experimental design: Group A received untreated drinking water; Group B received drinking water treated with 0.01–0.05% *v*/*v* Cid 2000™ (hydrogen peroxide, acetic acid and paracetic acid); Group C was challenged by *C. jejuni* and received untreated drinking water; and Group D was challenged by *C. jejuni* and received drinking water treated with 0.01–0.05% *v*/*v* Cid 2000™. The use of Cid 2000™ started on day 1 and was applied in intervals until the end of the experiment at 36 days, while the *C. jejuni* challenge was applied on day 18. Potential biomarkers were investigated in serum, feces, intestinal tissue, intestinal content, and liver samples of broilers. Statistical analysis revealed significant increases (*p* < 0.001) in serum cortisol levels in *C. jejuni*-challenged broilers. Serum fluorescein isothiocyanate dextran (FITC-d) increased significantly (*p* = 0.004) in broilers challenged by *C. jejuni* and treated with drinking water disinfectant, while fecal ovotransferrin concentration also increased significantly (*p* < 0.001) in broilers that received the drinking water disinfectant alone. The gene expression levels of *occludin* (*p* = 0.003) and *mucin-2* (*p <* 0.001) were significantly upregulated in broilers challenged by *C. jejuni*, while mucin-2 significantly increased in birds that were challenged and received the drinking water disinfectant (*p* < 0.001). *TLR-4* expression levels were significantly (*p =* 0.013) decreased in both groups that received the drinking water disinfectant, compared to the negative control group. Finally, the *C. jejuni* challenge significantly increased (*p =* 0.032) the crypt depth and decreased (*p* = 0.021) the villus height-to-crypt-depth ratio in the ileum of birds, while the tested disinfectant product increased (*p* = 0.033) the villus height in the jejunum of birds. Furthermore, the counts of *C. jejuni* in the ceca of birds (*p* = 0.01), as well as its translocation rate to the liver of broilers (*p* = 0.001), were significantly reduced by the addition of the water disinfectant. This research contributes to novel insights into the intricate interplay of water disinfection and/or *C. jejuni* challenge with potential intestinal biomarkers. In addition, it emphasizes the need for continued research to unveil the underlying mechanisms, expands our understanding of broiler responses to these challenges and identifies breakpoints for further investigations.

## 1. Introduction

Emerging biomarkers hold the potential to revolutionize veterinary medicine by enabling swift disease diagnosis, enhancing animal health monitoring, and optimizing welfare and production efficiency [1]. Nevertheless, the journey from biomarker identification to practical application is often fraught with challenges. Existing diagnostic tools for assessing gut health in poultry suffer from drawbacks, including invasiveness, subjectivity, labor intensiveness, and/or time consumption [2]. This underscores the urging need for noninvasive and objective diagnostic tools that enable the early detection of intestinal health issues throughout the production cycle [3]. Moreover, the availability of reliable biomarkers is crucial for understanding the pathogenesis of clinical and sub-clinical disorders, as well as for evaluating the efficacy of novel treatments against intestinal pathogens [2,4,5]. The identification of such biomarkers holds the potential to facilitate the implementation of preventive measures for enhanced surveillance and control within the farm.

Over the past decades, human infections caused by bacteria of the genus *Campylobacter* have emerged as the leading cause of bacterial foodborne gastroenteritis worldwide [6,7]. In particular, campylobacteriosis stands at the forefront of zoonotic diseases reported in the European Union, with over 137,000 documented cases in 2022 [8]. Poultry meat, particularly from broilers, constitutes the primary source of human infection, with *Campylobacter jejuni* being the predominant species involved in reported cases [8].

Increased surveillance and interdisciplinary collaborations, based on the “One Health” approach, have significantly contributed to identifying *Campylobacter* spp. reservoirs in nature and devising control strategies [9]. This approach integrates efforts across veterinary, medical, environmental, and other relevant disciplines to address *Campylobacter* transmission pathways comprehensively [9]. Particularly, Quantitative Microbial Risk Assessments for the presence of *Campylobacter* spp. in poultry meat have been used as a guidance tool and decision-making aid for controlling the presence of the microorganism throughout the production chain [10,11]. Although there are significant differences between countries in approaching these models, all risk assessments conclude that control *Campylobacter* spp. in primary production (at the farm level) is the most effective intervention measure to reduce the incidence of human infection [10,11,12,13].

Recently, the European Food Safety Authority (EFSA) proposed the addition of organic acids or biocidal mixtures based on chlorine or hydrogen peroxide to the drinking water of poultry as a measure to reduce the prevalence of *Campylobacter* spp. in broiler farms [10]. However, until now, no method for controlling the microorganism at the farm level has been proven to be fully effective and simultaneously economically viable for controlling *Campylobacter* spp. in broiler production [11]. On the other hand, there are several commercially available acid-based products used for water sanitation in poultry farms, that have been mentioned in previous in vitro studies for their anti-*Campylobacter* activity [14,15]. These products are used for the “deep cleaning” of the water supply system (when birds are absent) as well as for improving the quality of drinking water (when birds are present). Depending on their composition, some acid-based products have been approved as “additives” [16] as their ingredients improve the performance of poultry, while others are classified as “disinfectants” ensuring the quality of drinking water and controlling pathogenic microorganisms such as *Salmonella* spp. and *Campylobacter* spp. [14,15,17,18].

Contrary to the general assumption of *C. jejuni* as a commensal organism in broilers over the past decade, recent studies indicate a more complex relationship among *C. jejuni* and broiler gut health, making understanding its role in broiler health challenging [19]. To address this, objective and quantitative measurements of biological processes that can change in abundance under pathological conditions can play a crucial role [2]. These markers, which are found in various biological samples such as serum and fecal material, indicate inflammation, gut barrier leakage, or tissue damage [2,5,20].

However, gut health cannot be assessed by a single universal biomarker. Hence, there arises a necessity for a collection of objective biomarkers that can be identified in biological fluids and excreta. This initial step is crucial for enabling important assessments that are relevant to farm settings, in particular for the evaluation of intestinal inflammation and barrier function. Therefore, the present study aims to investigate various potential biomarkers in serum (IL-10, cortisol, FITC-d), feces (ovotransferrin), intestinal tissue (expression levels of *OCL*, *MUC2*, *TLR-4* and histomorphometry), alimentary content (*C. jejuni* caecal counts, microbiota composition), and liver (*C. jejuni*-translocation) of broilers subjected to continuous water disinfection (Cid 2000™) and/or challenged by *C. jejuni*.

## 2. Materials and Methods

### 2.1. Experimental Facilities, Biosecurity and Ethics

The experimental investigation was performed at the Aristotle University of Thessaloniki (AUTh), Greece, in the experimental facilities of the Unit of Avian Medicine at the School of Veterinary Medicine. The study adhered to the Council Directive (2010/63/EU) and Greek regulations concerning animal husbandry, euthanasia, experimental protocols, and biosecurity measures for research animals. Approval was obtained from the Ethical Committee of the School of Veterinary Medicine and the Greek Veterinary Authority.

Environmental parameters such as temperature, relative humidity, and lighting were monitored daily using HOBO UX100-003 Temperature/Relative Humidity data loggers (Onset Computer Corporation, Bourne, MA, USA) in each room. Adjustments were made based on recommendations from the breeding company (Aviagen^®^, Huntsville, AL, USA) [21]. Birds were provided ad libitum access to feed throughout the trial. To simulate commercial conditions, broilers were placed on a 5 cm thick layer of fresh wood shavings.

### 2.2. Experimental Design

**Figure 1:**
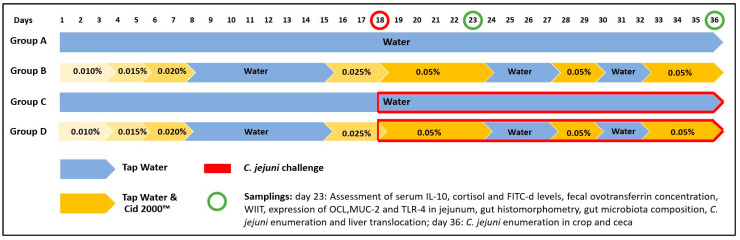
Experimental design and tested product dosage scheme.

A total of one hundred and forty-four (144) one-day-old broiler chicks (Ross 308^®^) were purchased from a local commercial hatchery. After arrival, the birds were randomly allocated into 4 treatment groups with four replicates (9 chicks per replicate), summing a total of 36 chicks per group, according to the following experimental design: group A = birds were not challenged and received drinking water without any treatment; group B = birds were not challenged and received drinking water treated with 0.01–0.05% *v*/*v* Cid 2000™; group C = birds which were challenged by *C. jejuni* and received drinking water without any treatment; group D = birds were challenged by *C. jejuni* and received drinking water treated with 0.01–0.05% *v*/*v* Cid 2000™. The entire duration of the experiment was 36 days, whereas the tested product was applied in the drinking water of broilers in groups B and D, in intervals (Figure 1).

### 2.3. Feed

To meet the nutrient requirements of the broiler chicks, three complete basal diets were formulated for the starter (1 to 13 days), grower (14–23 days) and finishing period (24 to 36 days), respectively. No antibiotic growth promoters, organic acids or essential oils were used. The composition of the three basal diets that were used during the experiment was analyzed utilizing a diode array near-infrared spectroscopy instrument (DA7250, PerkinElmer, Waltham, MA, USA). The analysis encompassed Weende components in addition to calcium and phosphorus. Detailed results can be found in Table S1.

### 2.4. Tested Product and Dosage Scheme

As a water supply, a 14 Lt container (tank) was filled daily with only fresh tap water (groups A and C) or water treated with Cid 2000™ (groups B and D). The commercial disinfectant, a blend of hydrogen peroxide, acetic and paracetic acids, was administered to the drinking water of broilers in groups B and D according to the schedule outlined in Figure 1.

Initially, during the first period of their life (1–8 days), the product was provided daily, with concentrations gradually increasing from 0.01% on day 1 to 0.02% on day 8, as recommended by the manufacturer. Subsequently, from the 9^th^ to the 15^th^ day, the birds consumed drinking water without the disinfectant. According to the manufacturer’s instructions, the dosage of the product may be increased during periods of stress. Therefore, building on this, two days prior to the challenge, the product was applied at a concentration of 0.025%, and from the challenge day until the day of sampling on the 23^rd^ day, the product was applied at the maximum dosage of 0.05%. Following this, from the 27^th^ to the 30^th^ day, the product was applied at the maximum dosage, and from the 32^nd^ to the 36^th^ day, it was again applied at the maximum dosage.

### 2.5. Challenge by Campylobacter jejuni

Broilers were challenged by *C. jejuni* on day 18. Prior to the study, analysis for *C. jejuni* was conducted to ensure the environment and the wood shavings used were free from *Campylobacter* contamination. The *C. jejuni* strain MB 4185 KC 40 was graciously provided by Professor Frank Pasmans from the Faculty of Veterinary Medicine, Ghent University, Merelbeke, Belgium. This strain, previously utilized in various studies [15,22,23,24] demonstrated high efficacy in colonizing broilers, establishing substantial populations within the gastrointestinal tract [24]. The strain was restored, and the final inoculum was prepared following the process that was previously described by Mantzios et al. (2023b) [15]. Finally, the birds were orally challenged using a plastic esophageal catheter, through which 1 mL of PBS containing approximately 6.5 × 10^6^ CFU/mL *C. jejuni* was administered.

### 2.6. Serum Biomarkers

#### 2.6.1. IL-10 and Cortisol Levels in the Serum

To measure the concentration of circulating IL-10 and cortisol, blood samples were obtained via jugular vein collection from 16 birds per group on the 23^rd^ day (5 days post-infection) by *C. jejuni*. Subsequently, serum was extracted by centrifugation at approximately 1000× *g* for 20 min. The obtained serum was then stored at −80 °C for further analysis. The concentration of IL-10 in the serum was quantified using a sandwich ELISA method, employing a commercial Chicken Interleukin 10 (IL10) ELISA Kit (DLR-IL10-Ch, Develop^®^, Wuxi, China), while the concentration of cortisol in the serum was measured by a commercial cortisol (cor) ELISA kit (DLR-Cortisol-Ge, Develop^®^, Wuxi, China) according to the manufacturer’s instructions and in accordance with previously reported methodologies [25,26,27]. The optical density (OD) at 450 nm was measured with an ELISA spectrophotometer (EnSpire Multimode Plate Reader, PerkinElmer, Waltham, MA, USA), and endogenous chicken IL-10 and cortisol concentrations were determined by referencing a standard curve constructed using known concentrations of chicken IL-10 and cortisol, respectively. The minimal detection limit of the IL-10 ELISA kit that was used in this study is 32 pg/mL, and for cortisol, it is 0.62 ng/mL, as stated by the manufacturers.

#### 2.6.2. Fluorescein Isothiocyanate Dextran (FITC-d)

On the 22^nd^ day of age, 3 birds per pen (12/group) were weighted and submitted to feed restriction for 12 h to induce a gut permeability challenge. Fluorescein isothiocyanate dextran (FITC-d, 100 mg, MW 4000; Sigma-Aldrich, St. Louis, MO, USA) was utilized to assess intestinal permeability, following the method described by Baxter et al. [4]. In particular, birds were orally administered FITC-d dissolved in phosphate-buffered saline (PBS). Three additional birds per group were gavaged by PBS only and were used as serum blank controls for each group, as previously outlined [4]. In each group, subsequently, after a 2 h duration, blood samples were collected from the jugular vein and left at room temperature for 3 h before undergoing centrifugation (500× *g* for 15 min) to obtain the serum. The serum was then diluted with PBS (1:1 PBS), and the presence of FITC-d in the serum was quantified using a multi-mode microplate fluorescence reader (Perkin-Elmer, Waltham, MA, USA) at an excitation wavelength of 485 nm and an emission wavelength of 528 nm. The standard curve was plotted according to absorption of standards prepared by spiking FITC-d at a range of concentrations (0–0.5 μg/mL). The amount of FITC-d in the serum for each bird was reported as µg of FITC-d per milliliter of serum.

### 2.7. Faecal Biomarkers

#### 2.7.1. Ovotransferrin

On the 23^rd^ day of age, pooled fecal samples were collected from each replicate of each experimental group to assess the concentration of ovotransferrin, as previously described by Goossens et al. [20]. The concentration of ovotransferrin in the fecal samples was determined by competitive ELISA (Chicken Ovotransferrin ELISA, KT-530, Kamiya Biomedical Company, Tukwila, WA, USA). A portion of 150 mg of the fecal samples was weighed and suspended in 1250 μL of TBS (50 mM Tris, 150 mM NaCl, pH = 7.2), in which 12.5 μL of protease inhibitor cocktail (P2714, Sigma-Aldrich) was added. After a good mixing by vortex (2 × 1 min), the samples were centrifuged at 13,000× *g* for 10 min at 4 °C. The supernatant was collected and diluted at 1:50 in the diluent (1×) provided by the kit. The ELISA was performed according to the instructions of the manufacturer. The optical density (OD) at 450 nm was measured, and fecal ovotransferrin concentrations were determined by referencing a standard curve constructed using known concentrations of chicken ovotransferrin. The minimal detection limit of the ovotransferrin ELISA kit that was used in this study is 6.25 ng/mL.

#### 2.7.2. Whole Intestinal Transit Time (WITT) of Barium Sulfate

The potential induction of intestinal dysmotility caused by continuous drinking water disinfection, the challenge by *C. jejuni*, or their combination, was assessed by the WITT of barium sulfate among the experimental groups. The WITT refers to the duration taken for barium sulfate, upon oral administration, to be initially expelled in the feces [28,29]. This observation was conducted at 5 days post-infection (23 days of age) using three birds randomly selected from each of the four replicates within each experimental group (totaling 12 birds per group). Specifically, each bird was initially placed in an individual carton box measuring 50 × 50 cm. Subsequently, each bird orally received 10 g of barium sulfate (BARILUX 965MG/G, GI powder, SANOCHEMIA Pharmazeutika GmbH, Neufeld an der Leitha, Austria), after which their fecal excretions were monitored every 10 min for a span of 6 h to detect the discharge of stained fecal droplets following the barium administration, as illustrated in Figure S1.

### 2.8. Biomarkers on Intestinal Tissue

#### 2.8.1. Occludin (OCL), Toll-like Receptor-4 (TLR-4), and Mucin-2 (MUC2)

On the 23^rd^ day of age, four birds from each replicate (16 per group) were euthanized. The jejunal lumen of each bird was washed with buffered peptone water (BPW) to remove fecal and other materials. Subsequently, jejunal mucosa was carefully scraped using the blunt edge of surgical knife blades, and the collected scrapings were promptly transferred into microtubes, flash-frozen in liquid nitrogen, and stored at −80 °C until analysis for the expression of *occludin* (*OCL*)*, Toll-like receptor-4* (*TLR-4*)*,* and *mucin-2* (*MUC2*) [30,31]. Total RNA isolation was performed using Trizol reagent (Invitrogen Life Technologies, Carlsbad, CA, USA) following the manufacturer’s protocol. The concentration and purity of the obtained total RNA were assessed using a Nanodrop ND-1000 spectrophotometer (Thermo Fisher Scientific Inc., Waltham, MA, USA). For cDNA synthesis, a cDNA synthesis kit was used (ProtoScript^®^ II First Strand cDNA Synthesis Kit, New England Biolabs, Ipswich, MA, USA) in which 1 μg of total RNA from each sample was utilized as per the manufacturer’s instructions. Quantitative PCR was employed to measure the relative mRNA levels of *TLR-4*, *MUC2*, and *OCL*. Applied Biosystems^®^ SYBR^®^ Green PCR Master Mix and the StepONEplus Real-Time PCR System (Thermo Fischer Scientific, Waltham, MA, USA) were utilized along with primers listed in Table 1. Each reaction mixture (20 μL) consisted of 10 μL SYBR Premix, 0.4 μL of forward and reverse primer (10 μΜ each), 2 μL cDNA and 7.2 μL ddH_2_O. Reactions were performed using the following program: 95 °C for 1 min followed by 40 cycles of 95 °C for 15 s and 60 °C for 30 s. For the relative quantification of gene expression, the formula RQ = 2^−ΔΔCt^ was used. The results were normalized to the corresponding housekeeping gene *GAPDH*. All primers were verified for their amplification efficiency and linearity. All reactions were performed in duplicates and each experiment included two non-template, negative controls for each primer used.

#### 2.8.2. Gut Histomorphology

At the 23^rd^ day of age, tissue samples were collected from the duodenum (proximal to duodenal flexure), jejunum (proximal to Meckel’s diverticulum), and ileum (proximal to ileo-cecal junction). Samples were fixed in 10% buffered formaldehyde (pH 7.3) for 48–72 h. Subsequently, coronal sections (4–6 μm thickness) were placed on glass slides and stained with hematoxylin and eosin (H&E) for morphometric analysis. Sections were examined and measured with a Nikon Eclipse 50i light microscope using a Nikon DS-5 ML1 digital camera. For the histomorphometric analysis of the intestinal samples, a total of 10 well-preserved, vertically oriented villi were selected and measured from each histological slice, along with their corresponding crypts. Villi height (VH) was measured from the apex of the villi to the top of the submucosa. Crypt depth (CD) was measured from the base upwards to the point where the crypt transitions into the villi. Villi width (VW) was measured at the midpoint of each villus. Additionally, the crypt/villi (VH/CD) ratio was calculated [32]. For each variable studied, ten replicate measurements were taken from every sample, and the average values were utilized for subsequent statistical analyses.

### 2.9. Biomarkers on Alimentary Content and Liver

At the age of 23 days (5 days post-infection) samples from the crop and ceca were collected in aseptic conditions, placed on ice and immediately transported to the lab, for the enumeration of *C. jejuni*, *Escherichia coli*, *Clostridium* perfringens, lactic acid bacteria and *Bifidobacterium* spp. [15,33]. Briefly, crop and cecal samples were weighed and placed in sterile stomacher bags with nine times their volume of Maximum Recovery Diluent (MRD, Oxoid, Basingstoke, UK). Subsequently, the samples were homogenized for 60 s using a Stomacher (Interscience, Saint Nom la Bretêche, France). From the initial homogenate, decimal dilutions were prepared in MRD-containing tubes.

The enumeration of *C. jejuni* in the crop and cecal samples was according to ISO 10272-2:2017 [34] with modifications. In particular, surface plating was performed from each dilution onto charcoal cefoperazone deoxycholate agar (mCCDA 10,409; Liofilchem^®^ S.r.l, Roseto degli Abruzzi, Italy) supplemented with CCDA selective supplement (81,037; Liofilchem^®^ S.r.l, Roseto degli Abruzzi, Italy). The plates were then sealed in jars and incubated for 48 h at 42 °C under microaerophilic conditions (85% N_2_, 10% CO_2_, 5% O_2_; Thermo Scientific™ Oxoid™ CampyGen™ 2.5 L Sachet, Oxoid, Basingstoke, UK). Following incubation, putative positive *C. jejuni* colonies were enumerated and confirmed using standard biochemical and molecular methods, including peroxidase reaction, direct microscopy, gram staining, and PCR, following procedures previously outlined by Mantzios et al. [15].

Lactic acid bacteria were enumerated according to ISO 15214:1998 [35] after inoculation on de Man, Rogosa and Sharpe agar (MRS, Biolab) and incubation at 37 °C for 72 h. The enumeration of *Bifidobacteria* was performed according to ISO 29981:2010 [36], where using the Bifidus Selective Medium (BSM, Sigma) after incubation at 37 °C for 72 h, the characteristic pink colonies were counted. *E. coli* counts were determined according to ISO 16649-2:2001 [37], using the ChromoBio^®^ TBX agar (Biolab, Budapest, Hungary) where the characteristic green colonies obtained after incubation for 24 h at 44 °C under aerobic conditions were counted. *C. perfringens* counts were determined on 5% sheep blood agar (Columbia blood agar, 01-034, Scharlau Chemie S.A., Barcelona, Spain) containing *C. perfringens* selective supplement (SR0093 Oxoid Ltd., Cambridge, UK) after incubation at 37 °C for 48 h under anaerobic conditions, where the wide, circular, transparent colonies with typical “target” hemolysis (an inner zone of L-hemolysis and an outer zone of partial hemolysis) were counted [38]. The anaerobic conditions for *C. perfringens* and *Bifidobacteria* were generated using Oxoid™ AnaeroGen™ 2.5 L Sachet and were confirmed using Anaerotest^®^ (Merck 1.15112). The results were reported as colony-forming units per gram (CFU/g) of the sample and expressed as log_10_ CFU/g for subsequent statistical analysis.

### 2.10. Statistical Analysis

The effect of the challenge by *C. jejuni* and the disinfection of the drinking water on gut health biomarkers of broiler chicks was analyzed with one-way ANOVA using SPSS 26.0 (BM SPSS Statistics for Windows, Version 25.0. Armonk, NY, USA: IBM Corp.) and GraphPad Prism (version 9.1.2 for Windows^®^, GraphPad Software, San Diego, CA, USA). For the analysis of IL-10, cortisol and FITC-d levels in the serum, the ovotransferrin levels in feces, the WITT, the relative expression of *OCL*, *MUC-2* and *TLR-4* in jejunum, the gut histomorphometry (VH, VW, CD CH/CD), the *C. jejuni* counts in the crop and ceca, and the cecal microbiota composition (lactic acid bacteria, *Bifidobacteria*, *E. coli* and *C. perfringens*), post hoc comparisons between treatments were investigated by Duncan tests. Finally, the analysis of *C. jejuni* translocation in the liver of broilers was performed by crosstabs and Khi-2 tests using SPSS 26.0 (BM SPSS Statistics for Windows, Version 25.0. Armonk, NY, USA: IBM Corp.). The average values including the standard error of the mean (SEM) were calculated for every examined parameter and presented in the tables. The level of significance was set at *p* ≤ 0.05, and a statistical trend was underlined for 0.05 < *p* ≤ 0.1.

## 3. Results

### 3.1. Serum Biomarkers

**Figure 2:**
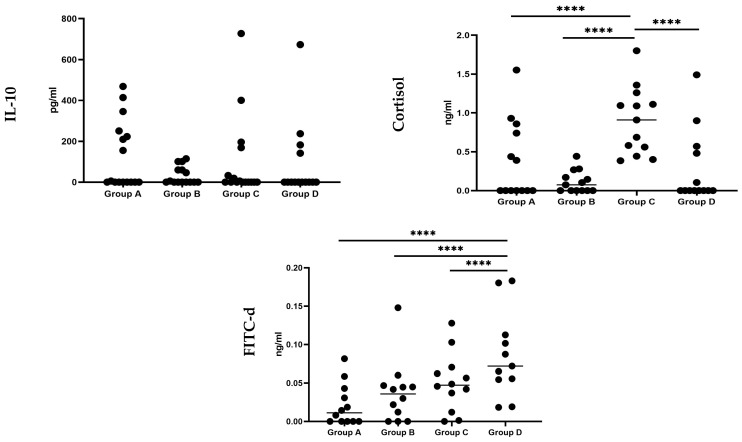
Effect of the drinking water disinfection on IL-10 (pg/mL), cortisol (ng/mL) and FITC-d (ng/g) levels in the serum of *C. jejuni* experimentally challenged broiler chicks. ****: differ significantly (*p* ≤ 0.05). Group A = birds were not challenged and received drinking water without any treatment; Group B = birds were not challenged and received drinking water treated with 0.01–0.05% *v*/*v* Cid 2000™; Group C = birds which were challenged by *C. jejuni* and received drinking water without any treatment; Group D = birds were challenged by *C. jejuni* and received drinking water treated with 0.01–0.05% *v*/*v* Cid 2000™.

The impact of the tested water treatment, the *C. jejuni* challenge, and their combination on the serum levels of IL-10, cortisol and FITC-d in broilers at day 23 of age, is illustrated in Figure 2 and Table S2. IL-10 protein levels in the serum of broilers did not differ significantly (*p >* 0.05) between the experimental groups. However, the serum cortisol levels of birds in group C were significantly (*p <* 0.001) higher compared to all the other experimental groups. Finally, the serum concentration of FITC-d in broilers exhibited a significant (*p <* 0.001) increase in group D compared to all other experimental groups.

### 3.2. Faecal Biomarkers

#### 3.2.1. Ovotransferrin

**Figure 3:**
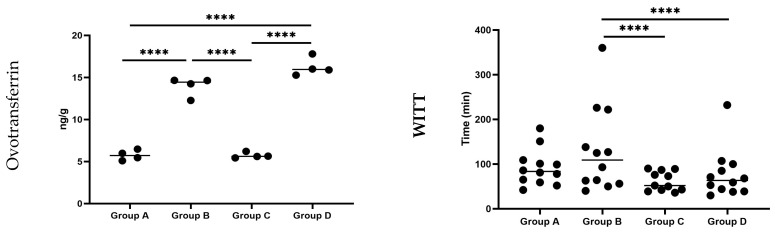
Effect of the drinking water disinfection on ovotransferrin levels in the feces (**left**) and the whole intestinal transit time (WITT) of barium sulfate (**right**) in *C. jejuni* experimentally challenged broiler chicks. ****: differ significantly (*p ≤* 0.05). Group A = birds were not challenged and received drinking water without any treatment; Group B = birds were not challenged and received drinking water treated with 0.01–0.05% *v*/*v* Cid 2000™; Group C = birds which were challenged by *C. jejuni* and received drinking water without any treatment; Group D = birds were challenged by *C. jejuni* and received drinking water treated with 0.01–0.05% *v*/*v* Cid 2000™.

The impact of the tested water treatment, the *C. jejuni* challenge, and their combination on ovotransferrin concentration in the feces of broilers at day 23 of age is illustrated in Figure 3 and Table S3.

Ovotransferrin was detected in the feces of birds from all the experimental groups in this study. Specifically, the concentration of ovotransferrin in the feces of birds in groups B and D was significantly higher (*p <* 0.001) compared to groups A and C. Furthermore, the concentration of ovotransferrin in the feces of birds of group D was also found to be significantly higher (*p <* 0.001) than that of group B.

#### 3.2.2. Whole Intestinal Transit Time (WITT) of Barium Sulfate

The impact of the tested water treatment, the *C. jejuni* challenge, and their combination in the WITT of barium sulfate broilers at day 23 of age is illustrated in Figure 3 and Table S4.

The mean passage time in experimental groups C and D was significantly lower (*p =* 0.05) compared to that of group B.

### 3.3. Biomarkers on Intestinal Tissue

**Figure 4:**
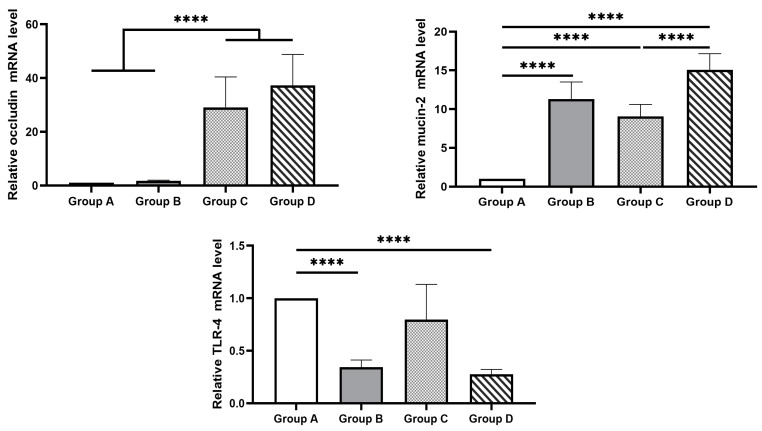
Effect of the drinking water disinfection on the relative expression of *OCL, MUC2* and *TLR-4* in jejunal segments of *C. jejuni* experimentally challenged broiler chicks (x ± SEM). ****: differ significantly (*p ≤* 0.05). Group A = birds were not challenged and received drinking water without any treatment; Group B = birds were not challenged and received drinking water treated with 0.01–0.05% *v*/*v* Cid 2000™; Group C = birds which were challenged by *C. jejuni* and received drinking water without any treatment; Group D = birds were challenged by *C. jejuni* and received drinking water treated with 0.01–0.05% *v*/*v* Cid 2000™.

The impact of the tested water treatment, the *C. jejuni* challenge, and their combination on the relative expression of *OCL*, *MUC2* and *TLR-4* in jejunal segments of broiler chicks at day 23 is illustrated in Figure 4.

Groups C and D exhibited significantly higher relative expression of *OCL* (*p =* 0.003) compared to the negative control group (group A) and group B. Regarding *MUC2* expression, experimental groups B, C and D exhibited significantly (*p <* 0.001) higher levels compared to the negative control group (group A). Additionally, the relative expression of *MUC2* in group D was significantly (*p =* 0.001) increased compared to group C. The relative expression of *TLR-4* was significantly (*p <* 0.001) decreased in groups B and D compared to group A.

**Figure 5:**
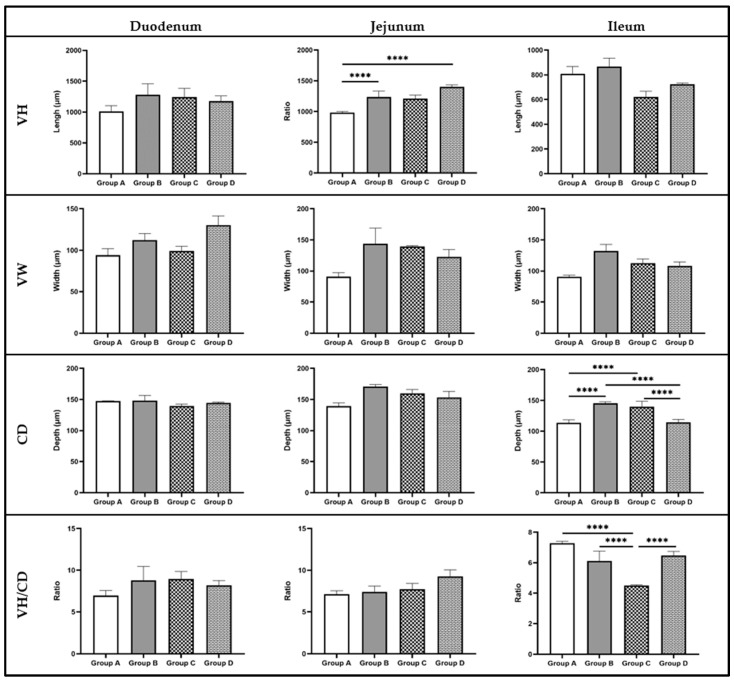
Effect of the drinking water disinfection on the gut (duodenum, jejunum, ileum) histomorphometry (VH, VW, CD, VH/CD) of *C. jejuni* experimentally challenged broiler chicks (x ± SEM). VH: Villus Height; VW: Villus Width; CD: Crypt Depth; VH/CD: Villus Height-to-Crypt-depth ratio. ****: differ significantly (*p ≤* 0.05).

The impact of the tested water treatment, *C. jejuni* challenge, and their combination on the histomorphometric analysis of the intestine (duodenum, jejunum, ileum) in broiler chicks at 23 days of age is illustrated in Figure 5, as well as in Table S5.

Statistical analysis and histomorphometric assessment of intestinal sections from various anatomical areas of the broilers’ gut revealed no significant (*p >* 0.05) differences among the experimental groups in the duodenum. However, significant effects (*p ≤* 0.05) were observed in the jejunum and ileum of broilers among groups. In particular, the villus height (VH) in the jejunum of birds in groups B and D was significantly increased (*p =* 0.033) compared to Group A. Concerning the ileum, the crypt depth (CD) in birds from groups B and C was significantly (*p =* 0.032) higher compared to groups A and D. In addition, the VH/CD ratio in experimental group C showed a significant decrease (*p =* 0.021) compared to the other groups. The VH in the ileum of birds in group C tended (*p =* 0.092) to be significantly lower compared to group B. Furthermore, the villus width (VW) in the ileum of group B tended (*p =* 0.066) to be significantly higher compared to group A.

### 3.4. Biomarkers on Alimentary Content and Liver

The impact of the tested water treatment, the *C. jejuni* challenge, and their combination on the microbiota composition and *C. jejuni* counts on the crop and the ceca of broiler chicks is illustrated in Table 2.

The results of the sampling tests performed on the 1^st^ day of the experiment were negative for the presence of *Campylobacter* spp. in the litter, experimental facilities/equipment, and chicks. *C. jejuni* was not detected in the content of crop and ceca of birds in groups A and B, highlighting the efficiency of the strict biosecurity measures that were applied during experimentation. After oral administration of *C. jejuni* to broilers at 18 day of age, considerable *C. jejuni* counts were enumerated in both the crop and ceca of birds at day 23 (5 days post-infection (dpi): *C. jejuni*-crop: 6.43 log_10_ CFU/g, *C. jejuni*-ceca: 7.84 log_10_ CFU/g), confirming the successful experimental infection. Disinfection of drinking water significantly (*p =* 0.010) reduced the *C. jejuni* counts in the ceca of birds by 0.74 log_10_ CFU/g. No significant (*p >* 0.05) differences in the counts of lactic acid bacteria in the crop of birds were observed between the different experimental groups. In addition, there were no significant (*p >* 0.05) differences in the counts of *C. perfringens*, lactic acid bacteria, *Bifidobacterium* spp., and *E. coli* in the ceca of birds across the study groups.

The effects of the tested water treatment, the *C. jejuni* challenge, and their combination on the translocation of *C. jejuni* in the liver are presented in Table 3.

*C. jejuni* was not detected in the liver of birds in groups A and B. When broilers were challenged with *C. jejuni*, there was a notable translocation of the microorganism to the liver, observed in 81.2% of the cases. The implementation of drinking water disinfection significantly reduced (*p =* 0.001) the translocation level to 37.5% in the liver of birds, marking a 43.7% decrease compared to group C.

## 4. Discussion

An ideal biomarker should exhibit a diverse set of characteristics, including sensitivity, specificity, reliability, robustness, stability, and minimal invasiveness [2,3]. In addition, the measurements of biomarkers should demonstrate minimal variability under healthy conditions, while showing an increase in response to alterations in gut health [39]. By possessing these qualities, an ideal biomarker can effectively serve as a reliable indicator of gut health status and aid in the diagnosis, monitoring, and management of gastrointestinal disorders in commercial poultry [2,3,5].

IL-10, a crucial anti-inflammatory cytokine, plays a pivotal role in modulating inflammatory responses, particularly within intestinal immunity, in poultry [40]. The intricate interplay of IL-10 in regulating inflammation underscores its importance in maintaining immune homeostasis, especially in the context of intestinal diseases such as necrotic enteritis and coccidiosis [26,27]. Cytokines are generally thought to be rapidly released during infection or other stimuli, remaining at basal levels under physiological conditions, and thus could be used as valuable markers [41]. Regarding *C. jejuni* infection, previous studies, including Humphrey et al. [42], Reid et al. [43], Mortada et al. [44] and Chagneau et al. [45], highlighted the dynamic expression of *IL-10* mRNA in tissue sections from various parts of the intestine of birds infected by *C. jejuni*. Particularly, Mortada et al. [44] reported that the challenge of broilers by *C. jejuni* did not significantly affect the *IL-10* mRNA levels in the ceca of broilers on 0, 1, 2, 3, and 7 dpi; however, an upregulation of *IL-10* was observed at 14 and 21 dpi [44]. Additionally, Chagneau et al. [45] observed a significant increase in cecal tonsil *IL-10* expression at 21 dpi, suggesting a potential correlation with long-term *C. jejuni* colonization. The results of our study indicate that IL-10 is not directly involved in the initial response during the first 5 dpi, which is in accordance with the previously mentioned studies. Recently, Chagneau et al. [45] reported the role of *C. jejuni* translocation in the increase of *IL-10* expression in the liver of the infected birds, suggesting one additional area for increased expression after challenge that could be linked with the increase in serum levels. Finally, the continuous drinking water disinfection using Cid 2000™ (counting at the sampling time: 23 continuous days) had no significant impact on IL-10 levels.

In the present study, oral infection of broilers by 10^6^ CFU *C. jejuni* KC40 at the 18^th^ day of age resulted in a significant increase in the serum cortisol levels at 5 dpi. Various studies across different avian species, such as domestic ducks, chicks and Japanese quails, have consistently reported increased levels of circulating cortisol and corticosterone in response to diverse stressors [46,47,48]. In instances of enteric stress or inflammation, signals from the intestinal epithelium, enteric muscles, and immune cells are conveyed to the brain via the central nervous system, particularly the vagus nerve [49]. This process leads to the release of cytokines from the intestine, activating the central nervous system and, consequently, the hypothalamic-pituitary-adrenal (HPA) axis, resulting in increased serum corticosterone levels. Elevated corticosterone modulates heterophile migration into the GI tract inflammation site to regulate inflammation and is also associated with sickness behavior in chicks due to increased cortisol secretion [50]. For *C. jejuni,* there was no previous study investigating the effects of the challenge of broilers on the serum levels of cortisol; thus, this study adds novel data to that field.

In-field observational studies suggest that the *C. jejuni* challenge has a significant effect on the flock behavior and overall welfare of broiler chicks [51,52,53]. Various factors have been proposed, including footpad dermatitis and arthritis, attributed to diarrhea and wet litter following the challenge [54]. On the other hand, fluctuations in corticosterone levels in chicks have been associated with discernible alterations in decision-making patterns, often referred to as ‘pessimism’ [55]. Birds treated with corticosterone exhibit an increased anticipation of punishment when confronted with ambiguous information, providing a behavioral indicator of potential welfare concerns in chicks [55]. Building on this, it is plausible to suggest that elevated cortisol levels in the serum of broilers after a *C. jejuni* challenge may contribute to the observed behavioral and welfare issues associated with the infection [51,52,53,54]. However, a more in-depth investigation is necessary to comprehensively assess this hypothesis, establishing a link between the *C. jejuni* challenge in broilers, increased cortisol levels in the serum, and subsequent behavioral alterations. Such research efforts should be conducted under both controlled experimental conditions and in real field settings to provide a thorough understanding of the interplay between *C. jejuni* infection and serum cortisol levels in broilers. Current in vitro studies suggest that stress-related hormones, particularly catecholamines, may contribute to the growth and virulence of *Campylobacter* [56]. Finally, the administration of Cid 2000™ demonstrated a noteworthy ability to normalize cortisol levels in infected chicks, exhibiting non-significant differences when compared to unchallenged birds. This observation suggests a potential ameliorative effect of Cid 2000™.

Various infectious and non-infectious agents, including feed structure, mycotoxins, *Salmonella* spp., *Eimeria* spp. and *Clostridium* spp., can compromise the intestinal barrier in chicks, leading to increased intestinal permeability and reduced performance [57]. Mild damage weakens the epithelial layer, resulting in fluid and ion loss into the intestinal lumen, while severe damage may lead to the translocation of microorganisms, toxins, and other molecules into the bloodstream [57].

Ovotransferrin, a 77.7 kDa glycoprotein, acts as an Acute Phase Protein (APP) in avian species. In chickens, its presence in the bloodstream can surge up to 300% during inflammation, effectively impeding microbial dissemination and modulating the activity of macrophages and heterophils [58]. Evaluating the loss of plasma proteins into the gastrointestinal tract has emerged as an indicator for assessing gut barrier integrity in birds afflicted with enteric ailments like necrotic enteritis and/or coccidiosis [20]. In this study, ovotransferrin was detected in fecal samples across all experimental groups. Prior proteome analyses have indicated that ovotransferrin may exhibit minimal expression levels in the epithelium and endothelium of the intestinal tract, even in healthy chickens, a fact corroborating our observations regarding concentrations detected in the negative control group [20,59]. Conversely, the notable increase in ovotransferrin concentration observed in the feces of birds in both groups treated with the drinking water disinfectant implies a potential compromise in the gut barrier integrity of these birds [20]. Furthermore, the combination of water disinfection and challenge by *C. jejuni* resulted in significantly higher levels of ovotransferrin in the feces of birds, indicative of an additive adverse effect.

FITC-d is another potential biomarker for intestinal integrity evaluation. It has a molecular weight of 3000–5000 Da and does not traverse intestinal epithelial tight junctions in high quantities unless the intestinal barrier is compromised [60]. It has been utilized in rat, mouse, and chicken studies to evaluate increased intestinal permeability and/or gut leakage [4,60,61,62,63]. In previous investigations, oral ingestion of FITC-d in broilers with necrotic enteritis [64] or/and *Eimeria* spp. [65] challenge resulted in a significant increase in the intestinal permeation of FITC-d molecules and their release in the bloodstream. In the current study, FITC-d concentration was significantly higher in the serum of birds that were both challenged and received the drinking water disinfection compared to all the other experimental groups. This suggests a potential utility of fecal ovotransferrin and FITC-d levels as a tool to evaluate gut barrier failure, albeit with variations in absolute levels between groups. Further research in healthy and diseased birds is required to establish accurate levels for their widespread application. Recently, Rysman et al. [3] reported that quantifying ovotransferrin in the colon can have merit for the evaluation of intestinal health in broilers under field conditions.

Previous research has shown that examining the gut passage time of barium sulfate as a biomarker provides an indication of intestinal motility [66]. In our investigation, in birds that were challenged and received the drinking water disinfectant, gut passage time was significantly reduced compared to those that received the drinking water disinfection alone. Previous research has noted an increase in passage time following *Eimeria* spp. infection, leading to a condition known as “gut stasis” [66]. Our findings highlighting the novel association between *C. jejuni* challenge and broiler intestinal motility represent a pioneering exploration in this field. Gaining insights into retention time and the specific duration of passage time across different gastrointestinal sections could further enrich our understanding, albeit this approach necessitates sacrificing animals and collecting digesta samples [29]. Notably, X-ray approaches are quite promising in this field [28,67].

The colonization of various pathogenic microorganisms and the presence of toxins lead to histomorphological changes in the intestine, resulting in subsequent effects on normal functioning and utilization of nutrients [68]. In the present study, the infection of birds with *C. jejuni* significantly increased crypt depth (CD) in the ileal region and markedly reduced the villus height-to-crypt-depth (VH/CD) ratio. Rzeznitzeck et al. (2022) reported that the CD in the intestines of turkeys (cecal region) may increase following experimental infection with *C. jejuni* and/or *C. coli* [69]. Given that the increase in CD is associated with enhanced regeneration of absorptive cells [70], in the current experimental group (positive control), this could indicate the initiation of epithelial repair mechanisms due to damages caused by the colonization of *C. jejuni*. Moreover, the reduction in the VH/CD ratio is linked to a smaller number of absorptive cells and a greater number of secretory cells responsible for mucus secretion [71]. Mucins constitute the main components of the mucous layer covering the intestinal epithelium [72], forming the first line of defense in the bird’s intestine. The presence of various pathogens, including *C. jejuni*, has been reported to induce mucin formation in the host’s intestinal epithelium [73], aiming to ensure the integrity of the epithelium against bacteria and/or their toxins. In this study, a significant increase in *MUC2* relative expression was observed in the jejunum of birds challenged by *C. jejuni*, in accordance with Ibrahim et al. [74].

It has been reported that organic acids, such as acetic and peracetic acid, contained in the product Cid 2000™, constitute a direct energy source for enterocytes. Therefore, their administration to poultry may contribute to the improvement of gut histomorphometry and consequently to the absorption of nutrients [75]. Specifically, the administration of organic acids, either through feed or drinking water, has been associated with an increase in villus height (VH), crypt depth (CD), and an enhancement in mucin composition [75,76]. In the present study, the administration of the commercial blend Cid 2000™ significantly increased VH in the jejunum and CD in the ileal region. Additionally, a trend towards increased villus width (VW) in the ileal area was observed (*p =* 0.066). Increased VH has been demonstrated to correlate with enhanced digestion and absorption, as well as increased activity of enzymes in the intestinal epithelium [32,71]. Furthermore, the elevation in VH is associated with increased mitotic activity, which is believed to enhance the absorptive capacity of the villi for various nutrients [77,78]. The increase in CD indicates intestinal mobilization for villus renewal, if necessary [79]. Thus, it could be suggested that the administration of the commercial product Cid 2000™ alone may enhance gut histomorphometry, consequently aiding in the better utilization of feed ingredients.

The administration of the commercial mixture Cid 2000™ also had a beneficial effect on the histomorphometry of infected birds’ intestines. Specifically, the product administration in infected birds significantly reduced crypt depth (CD) and increased the villus height-to-crypt-depth ratio (VH/CD) in the ileal region to such an extent that it did not significantly differ from the measurements in the ileum of non-infected birds. As previously mentioned, the VH/CD ratio is a significant parameter associated with the extent of the absorptive surface of the intestine. Furthermore, the increase in the VH/CD ratio is correlated with a reduction in the rate of intestinal epithelium renewal, resulting in improved absorption of nutrients and consequently improved bird performance. It is widely accepted that an increased frequency of intestinal epithelium renewal leads to greater maintenance needs, ultimately resulting in a slower growth rate in various animal species [80]. On the other hand, the reduction in the CD of the intestine leads to increased enzymatic activity, which further affects digestion and the utilization of the contents’ nutrients [81]. Therefore, the administration of the commercial mixture Cid 2000™ had an improving effect on the histomorphometry of the intestines of broilers infected with *C. jejuni*.

The infection of birds with *C. jejuni* led to its detection in the liver of birds, with a positivity rate of 81.2% in the total examined samples. The ability of *C. jejuni* to migrate from the intestine to other organs, such as the liver or spleen, has been previously observed [82,83]. Various mechanisms have been reported by which *C. jejuni* can breach the protective mucosal barrier of the intestine and enter the bloodstream. A primary mechanism appears to involve the microorganisms’ capability to alter the expression of genes or the structure of tight junction proteins, which constitute a crucial checkpoint for controlling paracellular permeability and cell cohesion of the intestinal epithelium [84,85]. Within the intricate intestinal epithelial cell barrier, occludin stands as a key component forming tight junctions [86]. In previous investigations, necrotic enteritis challenge in broilers notably influenced the expression of tight junction proteins, either in increasing or decreasing trends [86]. In our study, the challenge of broilers by *C. jejuni* significantly increased the relative expression level of *OCL* in the jejunum of birds compared to the unchallenged at 5 dpi. Disturbances in *OCL* expression have been associated with chronic inflammatory conditions such as Crohn’s Disease and ulcerative colitis in humans, underlining its significance in intestinal health [87].

It is well-known that increased intestinal permeability can lead to the passage of other microorganisms like *E. coli* and their toxins, transferring them to internal organs, and thereby prompting immune system activation [57,88]. Thus, assessing bacteria translocation in the liver can be used as a valuable marker for gut function and health in experimental settings [2]. The administration of the commercial acid mixture Cid 2000™ significantly reduced the isolation rate of *C. jejuni* from the livers of infected birds. This reduction might be attributed to the beneficial impact of the Cid 2000™ acid mixture on enteric histomorphometry, enhancing the immune system [89,90].

Generally, the defense against pathogens and maintenance of gut homeostasis relies on signaling pathways initiated by receptors such as toll-like receptors (TLRs). TLRs detect conserved microbial structures in the gut environment and trigger appropriate responses in eukaryotic cells [91]. Specifically, TLR2 and TLR4 predominantly recognize cell wall components of Gram-positive and Gram-negative bacteria. Activation of the transcription factor NF-κB is a key event downstream of TLR signaling, regulating immune and inflammatory responses [92]. In our study, we observed a downregulation of *TLR-4* expression in the groups treated with a drinking water disinfectant comprising a blend of organic and inorganic acids. Previous investigators reported that supplementation of broilers with organic acids can inhibit the activation of the TLR4/NF-κB signaling pathway and reduce the production of pro-inflammatory cytokines [93]. This suggests that the antimicrobial action of the commercial water disinfectant used in our study may have reduced the need for immune defense activity in the gut [91]. It is reported that organic acids can downregulate the *TLR-4* expression in the gut by alterations in the gut microbiome, either by reducing pathogenetic microorganisms such as *Clostridium* spp. [91,93] or by enhancing the growth of beneficial bacteria.

The gut microbiota plays a critical role in maintaining the health and performance of chickens, primarily by preventing the colonization of foodborne pathogens through competitive exclusion mechanisms [33]. This is achieved through various mechanisms, including nutrient provision, inhibition of pathogen adhesion to host cells, modulation of the host immune system, alteration of gut morphology, and production of organic acids and antimicrobial compounds. Therefore, assessing important bacterial genera in the intestinal content can serve as a valuable marker for gut health [2]. In our study, neither the challenge by *C. jejuni* nor the water disinfection altered the composition of the gut microbiota, as assessed by the presence of *E. coli*, *Clostridium* spp., *Lactobacillus* spp., and *Bifidobacterium* spp. However, further research is warranted to fully understand the impact of drinking water disinfection on gut immunity and microbiota.

The use of biomarkers in practical field applications necessitates the establishment of breakpoint values, mandating a comprehensive array of both in vivo and in-field studies. Beyond the immediate challenges related to sample collection and storage techniques, technical qualification is imperative for reliable biomarker assessment. Specifically, the susceptibility of ovotransferrin to degradation by fecal proteases imposes constraints, requiring the use of fresh material for accurate evaluation [20]. Moreover, to unravel the full mechanistic insights behind the mode of action of novel treatments, it is paramount to comprehend the baseline behavior of these biomarkers in the context of the disease [2].

Comparative studies, considering variations in protocols, genetic backgrounds of birds, strains used for infection, and dietary disparities, are indispensable [94]. These factors significantly influence outcomes and introduce variability, emphasizing the need for standardization to ensure robust and comparable data across studies. Addressing these considerations is pivotal for advancing the application of biomarkers in field settings and fostering their effective integration into poultry health management strategies [2,3].

## 5. Conclusions

In this comprehensive investigation, potential gut health biomarkers were evaluated to discern the multifaceted effects of continuous water disinfection (Cid 2000™), *C. jejuni* challenge, and their combination on broiler chicks’ gut health. Notably, disinfecting drinking water according to the dosage scheme employed in this study led to increased mean VH in the jejunum, CD in the ileum, and relative expression of *MUC2* in the jejunum. The challenge with *C. jejuni* significantly elevated serum cortisol levels, *MUC2* and *OCL* expression in the jejunum, increased CD and decreased the VH/CD ratio in the ileum. Disinfecting drinking water in broilers challenged by *C. jejuni* reduced CD and increased the VH/CD ratio in the ileum. However, it also further increased the relative expression of *OLC* and *MUC2*, along with serum concentration of FITC-d and fecal concentration of ovotransferrin compared to birds solely challenged by *C. jejuni*. This research contributes novel insights into the intricate interplay of biomarkers and gut health, emphasizing the need for continued investigations to unveil the underlying mechanisms and broaden our understanding of broiler responses to these challenges. By establishing critical breakpoints for future investigations, this study paves the way for deeper exploration into the intricate dynamics.

## Figures and Tables

**Table 1:** List of primers used for qRT-PCR.

Genes	Forward Primer	Reserve Primer	Fragment Size (bp)
*OCL*	5′-GAGCCCAGACTACCAAAGCAA-3′	5′-GCTTGATGTGGAAGAGCTTGTTG-3′	68
*TLR-4*	5′-CAGCACCAACTTCTCAGTTCC-3′	5′-TCTGCAGCCACACATTCTTT-3′	63
*MUC2*	5′-CCACACACCTGCCTACATGAA-3′	5′-GGATGGCAAGAGGACATATCAAA-3′	102
*GAPDH*	5′-CTGGCAAAGTCCAAGTGGTG-3′	5′-CCCTTGAAGTGTCCGTGTGT-3′	100

**Table 2:** Effect of the drinking water disinfection on the microbiota composition in crop and ceca of *C. jejuni* experimentally challenged broiler chicks (x ± SEM).

Bacterium	Group A (Negative Control)	Group B (Cid 2000™)	Group C (*C. jejuni*)	Group D (Cid 2000™ and *C. jejuni*)	*p* Value
*Crop*					
*C. jejuni*	ND	ND	6.43 ± 0.25	6.39 ± 0.10	0.880
Lactic acid bacteria	8.45 ± 0.09	8.57 ± 0.09	8.80 ± 0.11	8.46 ± 0.18	0.170
*Ceca*					
*C. jejuni*	ND	ND	7.84 ± 0.12 ^b^	7.10 ± 0.23 ^a^	0.010
*E. coli*	6.33 ± 0.16	6.35 ± 0.21	6.51 ± 0.23	6.00 ± 0.16	0.302
*C. perfringens*	1.38 ± 0.22	1.48 ± 0.18	1.44 ± 0.19	1.36 ± 0.15	0.966
Lactic acid bacteria	7.13 ± 0.21	7.10 ± 0.22	7.46 ± 0.22	7.40 ± 0.17	0.508
*Bifidobacterium* spp.	6.46 ± 0.14	6.52 ± 0.16	6.66 ± 0.08	6.62 ± 0.18	0.747

^a,b^ Means in the same row with a different superscript differ significantly (*p ≤* 0.05). ND: Not detected.

**Table 3:** Effect of the drinking water disinfection on the translocation (%) of *C. jejuni* in the liver of *C. jejuni* experimentally challenged broiler chicks.

*C. jejuni*	Group A (Negative Control)	Group B (Cid 2000™)	Group C (*C. jejuni*)	Group D (Cid 2000™ and *C. jejuni*)	*p* *(Chi2)*
Not detected	100%	100%	18.8%	62.5%	
Detected	0%	0%	81.2%	37.5%	
Total	100%	100%	100%	100%	0.001

Kruskal Wallis test: Group C vs. Group D: *p =* 0.007, Group C vs. Groups A, B: *p =* 0.021, Group D vs. Groups A, B: *p <* 0.001.

## Data Availability

None of the data presented were deposited in an official repository.

## Supplementary Materials

The following supporting information can be downloaded at: https://www.mdpi.com/article/10.3390/pathogens13050356/s1, Figure S1. Discharge of white-stained fecal droplets (indicated by the red arrows) following the barium sulfate administration for the determination of the WITT in broiler chicks.; Table S1. Analysis of the starter, grower, and finisher diets; Table S2. Effect of the drinking water disinfection on IL-10 (pg/mL), cortisol (ng/ml) and FITC-d (ng/g) levels in the serum of *C. jejuni* experimentally challenged broiler chicks (x ± SEM); Table S3. Effect of the drinking water disinfection on ovotransferrin concentration (ng/g) in the feces of *C. jejuni* experimentally challenged broiler chicks (x ± SEM); Table S4. Effect of the drinking water disinfection on the whole intestinal transit time (WITT) of barium sulfate in *C. jejuni* experimentally challenged broiler chicks (x ± SEM); Table S5. Effect of the drinking water disinfection on the gut (duodenum, jejunum, ileum) histo-morphometry of *C. jejuni* experimentally challenged broiler chicks (x ± SEM).
