# Supplementary material for: Investigation of Potential Gut Health Biomarkers in Broiler Chicks Challenged by Campylobacter jejuni and Submitted to a Continuous Water Disinfection Program
Source: Pathogens. 2024 Apr 26;13(5):356. doi: 10.3390/pathogens13050356 (PMC11124259; doi:10.3390/pathogens13050356)
Supplement: Supplementary file 1 [file pathogens-13-00356-s001.zip › pathogens-2925904-supplementary.pdf]

pathogens

MDPI

Article

Investigation of potential gut health biomarkers in broiler chicks challenged by *Campylobacter jejuni* and submitted to a continuous water disinfection program

Tilemachos Mantzios [1,2]*, Despoina E. Kiousi [2], Georgia D. Brellou [3]*, Georgios A. Papadopoulos [4], Vangelis Economou [5], Marili Vasilogianni [6], Elisavet Kanari [2], Evanthia Petridou [7], Ilias Giannenas [8], Guillermo Tellez-Isaias [9], Aglaia Pappa [2], Alex Galanis [2] and Vasilios Tsiouris [1].

Table S1. Analysis of the starter, grower, and finisher diets.

Calculated analysis (g/kg)	Starter diet (1st-13th day)	Grower diet (14th-23rd day)	Finisher (24th-36th day)
Moisture	11.11	10.87	10.95
Crude protein	21.84	20.62	17.83
Crude fat	5.52	6.58	6.96
Crude fiber	2.64	2.86	2.96
Starch	38.13	36.79	38.76
Crude Ash	9.44	9.80	8.98
Sugars	4.16	4.31	4.45
Calcium	1.31	1.57	1.68
Phosphorus	0.57	0.51	0.46
Energy (Mj/kg)	3.000	3.075	3.150

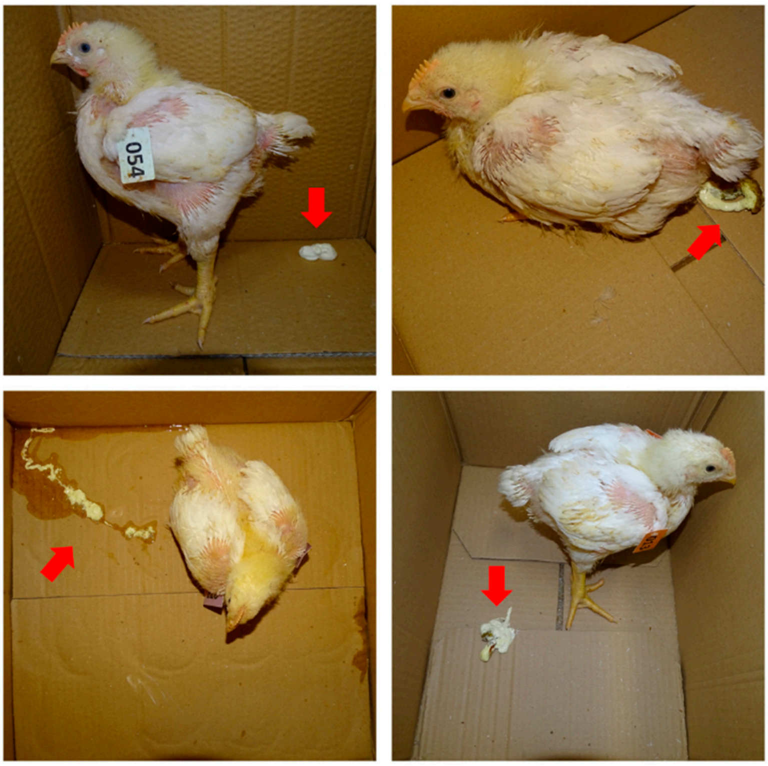

Figure S1. Discharge of white-stained fecal droplets (indicated by the red arrows) following the barium sulfate administration for the determination of the WITT in broiler chicks.

Table S2. Effect of the drinking water disinfection on IL-10 (pg/ml), cortisol (ng/ml) and FITC-d (ng/g) levels in the serum of *C. jejuni* experimentally challenged broiler chicks ($\bar{x}$ ± SEM).

**Serum biomarker**	**Group A (Negative control)**	**Group B (Cid 2000™)**	**Group C (*C. jejuni*)**	**Group D (Cid 2000™ & *C. jejuni*)**	***P value***
**IL-10** (pg/ml)	129.63±41.99	32.55±11.33	96.93±50.33	88.18±49.93	*0.278*
**Cortisol** (ng/ml)	0.38±0.14[a]	0.11±0.04[a]	0.90±0.12[b]	0.27±0.13[a]	*<0.001*
**FITC-d** (ng/g)	0.02±0.01[a]	0.04±0.01[a]	0.05±0.01[a]	0.09±0.02[b]	*0.004*

[a,b] Means in the same row with a different superscript differ significantly ($p \leq 0.05$). IL-10: Interleukin-10; FITC-d: Fluorescein isothiocyanate dextran

Table S3. Effect of the drinking water disinfection on ovotransferrin concentration (ng/g) in the feces of *C. jejuni* experimentally challenged broiler chicks ($\bar{x}$ ± SEM).

**Faecal biomarker**	**Group A (Negative control)**	**Group B (Cid 2000™)**	**Group C (*C. jejuni*)**	**Group D (Cid 2000™ & *C. jejuni*)**	***P value***
**Ovotransferrin** (ng/g)	5.75±0.30[a]	13.95±0.57[b]	5.72±0.17[a]	16.25±0.54[c]	*<0.001*

[a,b,c] Means in the same row with a different superscript differ significantly ($p \leq 0.05$).

Table S4. Effect of the drinking water disinfection on the whole intestinal transit time (WITT) of barium sulfate in *C. jejuni* experimentally challenged broiler chicks ($\bar{x}$ ± SEM).

**Faecal biomarker**	**Group A (Negative control)**	**Group B (Cid 2000™)**	**Group C (*C. jejuni*)**	**Group D (Cid 2000™ & *C. jejuni*)**	***P value***
**WITT** (in minutes)	91.92±11.65[a,b]	130.33±27.64[b]	61.55±6.51[a]	77.17±15.76[a]	*0.050*

[a,b] Means in the same row with a different superscript differ significantly ($p \leq 0.05$).

Table S5. Effect of the drinking water disinfection on the gut (duodenum, jejunum, ileum) histomorphometry of *C. jejuni* experimentally challenged broiler chicks ($\bar{x} \pm$ SEM).

**Parameter**	**Group A (Negative control)**	**Group B (Cid 2000™)**	**Group C (*C. jejuni*)**	**Group D (Cid 2000™ & *C. jejuni*)**	***P value***
			Duodenum		
VH	1011.30±92.92	1283.16±173.63	1240.98±142.28	1175.71±87.37	*0.532*
VW	93.94±7.96	112.30±7.92	99.10±5.71	130.07±11.48	*0.125*
CD	147.51±0.49	148.37±8.15	139.70±2.82	144.83±0.95	*0.550*
VH/CD	6.96±0.62	8.80±1.65	8.95±0.90	8.19±0.59	*0.568*
			Jejunum		
VH	982.48±19.25^{a}	1237.77±94.65^{b}	1208.90±60.14$^{a.b}$	1401.03±31.84^{b}	*0.033*
VW	90.86±6.31	143.62±25.52	139.19±1.63	122.62±11.78	*0.177*
CD	139.27±5.26	170.73±3.18	159.67±6.29	153.26±9.63	*0.105*
VH/CD	7.11±0.42	7.41±0.70	7.72±0.71	9.26±0.77	*0.248*
			Ileum		
VH	807.99±59.18$^{a.b\#}$	866.18±67.73$^{b\#}$	622.48±44.04$^{a\#}$	724.29±8.51$^{a.b\#}$	*0.092*
VW	90.71±2.29$^{a\#}$	132.26±10.62$^{b\#}$	112.39±6.97$^{a.b\#}$	108.02±6.63$^{a.b\#}$	*0.066*
CD	113.40±5.18^{a}	145.41±2.37^{b}	139.81±8.71^{b}	114.35±4.58^{a}	*0.032*
VH/CD	7.28±0.12^{b}	6.12±0.64^{b}	4.50±0.04^{a}	6.47±0.27^{b}	*0.021*

^{a,b} Means in the same row with a different superscript differ significantly ($P \leq 0.05$). $^{\#}$ Means in the same row with a different superscript tend to differ significantly ($P \leq 0.10$). VH: Villus Height (μm); VW: Villus Width (μm); CD: Crypt Depth (μm); VH/CD: Villus Height to Crypt depth ratio.
